# Immunomodulatory Effect of *Gymnema sylvestre* (R.Br.) Leaf Extract: An *In Vitro* Study in Rat Model

**DOI:** 10.1371/journal.pone.0139631

**Published:** 2015-10-16

**Authors:** Vineet Kumar Singh, Padmanabh Dwivedi, B. R. Chaudhary, Ramesh Singh

**Affiliations:** 1 Centre of Advanced Study in Botany, Banaras Hindu University, Varanasi, U.P., India; 2 Department of Plant Physiology, Institute of Agricultural Sciences, Banaras Hindu University, Varanasi, U.P., India; 3 Department of Zoology, Udai Pratap Autonomous College, Varanasi, U.P., India; Institut National de la Santé et de la Recherche Médicale (INSERM), FRANCE

## Abstract

*Gymnema sylvestre* Wild R.Br (family: Asclepidaceae) is a valuable medicinal plant used in folk medicine to treat diabetes, obesity, asthma etc. in India for antiquity. Diabetes mellitus is a syndrome characterized immunologically by lymphocyte apoptosis and reduced cell-mediated and humoral immunity. Modulation of immune responses to alleviate diseases has been of interest, and traditional herbal medicines may play an important role in this regard. In this study, we aim to evaluate the immunomodulatory potential of methanolic extract of *G*. *sylvestre* leaf using rat model. HPLC analysis of leaf extract was carried out for gymnemic acid. The method involves the initial hydrolysis of gymnemic acids, the active ingredients, to a common aglycone followed by the quantitative estimation of gymnemagenin, using gymnemagenin as reference standard. Gymnemic acid content was 2.40% (w/w) in *G*. *sylvestre* leaf extract. *In vitro* immunomodulatory activity of the methanolic extract of *G*. *sylvestre* leaf (1–200μg/ml) was evaluated by gauging its effects on nitroblue tetrazolium reduction and nitrite release in rat peritoneal macrophages and on mitogen (ConA, PHA and LPS) induced splenic lymphocyte proliferation. *G*. *sylvestre* leaf extract showed significant (<0.05) enhancement in NO and ROS generation in macrophages and in proliferation of lymphocytes in dose dependent manner. EC_50_ value was 3.10, 3.75 and 2.68μg/ml for NBT reduction, nitrite release and lymphoproliferation, respectively. Potential effect was observed at 100 μg/ml in NO and ROS generation in macrophages and 20 μg/ml in lymphocyte proliferation. *G*. *sylvestre* leaf extract stimulates macrophage reactivity, increasing the level of activity even higher when combined with PMA or LPS. These findings suggest the presence of active compounds, gymnemic acid, in methanolic extract of *G*. *sylvestre* leaf that stimulates both myeloid and lymphoid components of immune system, and therefore can restore the innate immune function. Through this study, the traditional knowledge of anti-diabetic property of *G*. *sylvestre* is scientifically supplemented with its immunomodulatory properties.

## Introduction

Immune system dysfunction is responsible for pathophysiology of many diseases. Modulation of immune responses, either by stimulation of the immune system or by suppression of undesired immune reactions, to alleviate such diseases has been of great interest for years. Immunomodulatory therapy could provide an alternative to conventional chemotherapy for a variety of diseased conditions, especially when host’s defense mechanisms have to be activated under the conditions of impaired immune responsiveness.

The knowledge of immunomodulatory properties and mechanisms of action of herbal medicines are wanted not only for the discovery of novel therapeutic agents that would validate folkloric remedies, but also to provide new insights into immune function and possible avenues of immunotherapy. Immunopharmacology, a new developing branch, aims at searching for immunomodulators, and many researchers have focused on identifying plant derived natural substances that can serve as immunomodulators to control the outcome of certain immune responses.

Many herbs, such as *Achyrocline alata* [1], *Asparagus racemosus* [2], *Azadirachta indica* [3], *Centella asiatica* [4], *Chenopodium ambrosioides* [5], *Clausena excavata* [6–8], *Coriolus versicolor* [9], *Curcuma longa* [10], *Enicostema axillare* [11], *Phyllanthus debelis* [12], *Pouteria cambodiana* [13,14] and *Tinospora cordifolia* [15–17] have been shown to alter the immune function and to possess a wide arrays of immunomodulatory effect in rat and mice. Immunomodulatory agents of plant origin increase the immune responsiveness by activating competent cells of immune system, and systematic studies on medicinal plants are the need to substantiate prophylactic and therapeutic claims made regarding their clinical utility [18]. *Viscum album*, a traditional phytomedicine of Europe, extract preparations are extensively used as complementary therapy in cancer and also in treatment of several inflammatory pathologies. An *in vitro* study has suggested no risk of safety expected from the exposition of cancer cells to chemotherapeutic drugs and *Viscum album* extract simultaneously [19]. Dr. Kaveri’s group at Paris, France, has studied various cellular and molecular approaches to define mechanistic basis for the therapeutic benefit of *Viscum album* preparations, and they revealed that these preparations exert anti-tumor activities, which involve cytotoxicity, inhibition of angiogenesis associated with induction of apoptosis of endothelial cells, anti-inflammatory effect by inhibiting cytokine-induced PGE_2_ secretion via selective inhibition of Cyclo-oxygenase-2 and other immunomodulatory mechanisms [20–30].

Type 2 diabetes is a syndrome characterized by impaired carbohydrate, fat and protein metabolism due to endocrine disorder. Immunologically, lymphocyte apoptosis [31] and depression of cell-mediated and humoral immunity have been reported in diabetics [32, 33]. *Gymnema sylvestre* R.Br (Family, Asclepidaceae) is a well known and highly valuable medicinal plant used in folk medicine to treat diabetes, obesity, asthma etc. in India for antiquity [34]. Ayurvedic anti-diabetic formulations marketed in India contain mainly *G*. *sylvestre*. The close relation between endocrine and immune system, diabetes induced lymphocyte apoptosis [31] and depression of cell mediated and humoral immunity [32, 33], and anti-diabetic activity of medicinal plants related with their antioxidant property [35] have led to the assumption that *G*. *sylvestre* leaf extract affecting the endocrine correlates of fat and glucose metabolism [36–41] may also be involved in modulation of immune function. Hence, in this study, we aim to assess the immunomodulatory activity of the methanolic extract of *G*. *sylvestre* leaf *in vitro* on rat peritoneal macrophage responses and splenic lymphocyte proliferation.

## Materials and Methods

### Chemicals

Phorbol myristate acetate (PMA), tetrazolium dyes, NBT (Nitroblue Tetrazolium) and MTT [3-(4, 5-dimethylthiozol-2-yl)-2, 5 diphenyl tetrazolium bromide], mitogens [concanavalin A (Con A), phytohemaglutinin (PHA) and lipopolysaccharide (LPS)] and Culture medium (RPMI-1640) were procured from Sigma, USA. Antibiotic-Antimycotic solution, L-glutamine, fetal bovine serum (FBS), lymphocyte separation medium (Hisep), dimethyl sulfoxide (DMSO) were purchased from Himedia Laboratories Pvt. Ltd. (India). The culture medium was supplemented with 5% heat inactivated FBS, Antibiotic-Antimycotic solution, 10mM HEPES and 50μM Mercaptoethanol. All other chemicals and solvents used were analytical grade.

### Herbal Plant

*G*. *sylvestre* R.Br (Family, Asclepidaceae: Botanical synonym, *Asclepias geminata* Roxb. and *Periploca sylvestres* Retz.), commonly known as Periploca of the woods (English) and Gurmar (Hindi) meaning sugar destroying, is a woody liana native to tropical forests of central and southern India, parts of Asia and tropical Africa. Fresh leaves of *G*. *sylvestre* were collected from Rajiv Gandhi South Campus of Banaras Hindu University (BHU) at Barkachha, Mirzapur (25.15°N:82.60°E). The voucher specimen was prepared, authenticated and deposited in the herbarium section of Botanical Survey of India, Central Regional Centre at Allahabad, Uttar Pradesh, India (Ref. No.VKS-1).

#### Preparation of leaf extract

The collected leaves were washed with distilled water, shade dried, powdered, and stored in an airtight container until further use. Dried powder (20 g) was soaked in 200ml 70% methanol for two days at room temperature (25±1°C) with occasional stirring. After two days, extract was filtered, and the residue again soaked in same amount of methanol for another two days and then filtered. The pooled hydro methanolic extract (GS extract) was rotary evaporated under reduced pressure below 40°C and stored at –20°C. For *in vitro* culture experiments, GS extract was dissolved in DMSO and further diluted with PBS and culture medium; the final concentration of DMSO in assay culture was < 0.1% (v/v).

#### Analysis of extract

The main constituent of *G*. *sylvestre* is a mixture of 17 oleanane type triterpenoid saponins known as gymnemic acids [34, 42]. Since non-availability of the different reference standards is difficult, the estimation of gymnemic acids is performed by hydrolysing the extract first with alkali and then with acid. Gymnemagenin thus obtained is estimated by HPLC, and the total gymnemic acid is calculated by applying the molecular weight corrections. Analysis of *G*. *sylvestre* leaf extract was carried out at Natural Remedies Pvt. Ltd., Bangalore, India. Reference standard, gymnemagenin, 10 mg was dissolved in 20 ml HPLC grade methanol by sonicating for 5 min, cooled and made the final volume up to 50 ml with methanol. Sample for HPLC was prepared using acid-base hydrolysis. Briefly, 1g of fine leaf powder was dissolved in 30 ml of 50% (v/v) methanol. To the solution, 2 ml of 12% (w/v) KOH was added and refluxed on a boiling water bath for 1 hour. The solution was cooled, 5.5 ml 4N HCl was added and again refluxed on a boiling water bath for 1 hour. Following cooling, the solution was centrifuged, supernatant transferred, and pH was adjusted to 7.5–8.5 with 12% KOH. Final volume was made to 100 ml with 50% methanol, filtered through membrane filter and subjected to HPLC analysis.

Chromatographic separation was achieved on column, Luna 5μ C-18(2) size; 250 x 4.60mm (Phenomenex). Mobile phase consisted of phosphate buffer (1mM KH_2_PO_4_ and 0.05% Orthophosphoric acid) as solvent A and Acetonitrile as solvent B. Gradient elution programme was used: from 0 to 20 min, 25% solvent B; from 20 to 25min, 55% solvent B; from 25 to 30 min, 60% solvent B; from 30 to 35min, 60% solvent B; from 35 to 40min, 25% solvent B. The flow rate was 1.5 ml/min. Reference standard 20 μl was injected, and chromatogram was recorded. Same was repeated thrice, and mean area and % relative standard deviation (RSD) were calculated (RSD<1%). 20 μl of sample extract was injected, and chromatogram was recorded. Amount of gymnemagenin (Mol.wt. 506.70) was converted into amount of gymnemic acid (Mol.wt. 809.00), and gymnemic acid (GA) content was calculated by following formula:

$$\text{GA content}\;(\%\;\text{w}/\text{w})\;=\;\frac{\text{Area of sample}}{\text{Area of standard}}\times \frac{\text{wt}.\;\text{of standard}\;(\text{mg})}{\text{Standard dilution}\;(\text{ml})}\times \frac{\text{Sample dilution}\;(\text{ml})}{\text{wt}.\;\text{of sample}\;(\text{mg})}\times \frac{809.00}{506.70}\times \text{Purity of standard}\;(\%)$$

### Animals

Wister rats, in-house bred at Institute of Medical Sciences, Banaras Hindu University, Varanasi, were procured and used in this study. Animals of 2 to 3 month old with body weight ranging from 200 to 250 g were selected and acclimatized to laboratory conditions with controlled temperature (25±1°C). Rats were housed in standard polypropylene cages (size: 41 x 28 x 14 cm) with stainless steel top grill having facilities for pellet food and drinking water glass bottle. The cages had rice husk bed (about 2 cm thick). Rats were fed with standard pellet food *ad libitum*. Aquaguard (Eureka Forbes Ltd., Bombay, India) filtered water was provided to animals in glass bottles with stainless steel sipper tubes *ad libitum*. Sexual dimorphism in immune function is reported in vertebrates [43, 44]; hence, only males were included in study. In an experiment, peritoneal macrophages were harvested from 3–4 rats and pooled for each *in vitro* assay, NBT reduction and Nitrite assay. The spleen of these rats was harvested for lymphoproliferation assay. Splenocyte suspensions were not pooled, to avoid mixed lymphocyte reaction. Three different independent experiments were carried out.

#### Ethics Statement

The guidelines of the Institutional Ethics Committee and the Committee for the Purpose of Control and Supervision of Experiment on Animals (CPCSEA), Ministry of Environment, Forest and Climate Change, Government of India, were followed. The study was approved by Central Animal Ethical Committee of Banaras Hindu University, Varanasi (Ref. No. Dean/12-13/CAEC/29).

Following acclimation, rats were injected intraperitoneally with 2ml FBS as stimulant to elicit peritoneal macrophages. After 72 h, rats were anesthetized with barbiturate (i. p. injection of Nembutal; 40 mg/kg body wt.). The peritoneal exudates were harvested by lavage with culture medium. Following lavage, spleen was excised. Rats were euthanized by barbiturate overdose followed by harvest of lungs. The carcasses were buried under the soil. The peritoneal exudates were centrifuged at 400xg for 30 min at 8°C, and cell pellet was washed with 0.2 M PBS (pH 7.2) twice and re-suspended in complete culture medium. The cell number was determined in a hemocytometer, and cell viability (> 90%) was tested by trypan-blue dye exclusion technique. Cell density was adjusted to 5×10^6^ cells/ml.

#### Preparation of splenic lymphocyte suspension

Following peritoneal lavage, the excised spleen was weighed, and splenic cell suspension was prepared following method of Tripathi et al [45, 46]. Briefly, under aseptic conditions, spleen was immediately pressed and flushed through a nylon strainer of pore size <100 μm into complete culture medium (5 ml/spleen) to get single cell suspension. Splenic cell suspension was treated with hemolysate buffer, washed with PBS twice and re-suspended in complete culture medium. Splenic lymphocytes were isolated by density gradient centrifugation using lymphocyte separation medium, HiSep (Density 1.077 g/ml). Lymphocytes were counted in hemocytometer and assessed for viability by trypan blue exclusion test, and viable cells (> 90%) density was adjusted to 2×10^7^ cells/ml.

### *In vitro* assays

#### NBT reduction assay

NBT reduction assay was performed following the method of Berger and Slapnickova [47]. Briefly, macrophage cell suspension was seeded (10^5^ cells/ well) in a flat bottom 96-well plate (Nest^®^ Biotech., China). PMA or LPS, inducing respiratory burst, were added into well to give final concentration of 1μg/ml in positive control culture. GS extract was added (final concentration: 1, 5, 10, 20, 50, 100 and 200 μg/ml) into wells with or without PMA and LPS, and final volume was made to 100 μl with medium. Macrophage cell suspension without any stimulant, PMA, LPS or GS extract, in well represented untreated control. Additional well containing 100 μl of culture medium served as blank. All assays were made in triplicate.

Plate was incubated in humidified CO_2_ atmosphere at 37°C for 24 h. After incubation, 50 μl of RPMI containing NBT (1 mg/ml) was added to each well and further incubated as above for 2 h, centrifuged at 700xg, washed with PBS and fixed in 70% methanol. Twenty microlitre of 0.1% Triton X-100 was mixed in each well, and the formazan crystals were dissolved by mixing 120 μl KOH (2 M) and 140 μl DMSO. Optical density (OD) was measured at 620 nm with the help of ELISA plate reader (Thermo Multiscan). OD was corrected by subtracting the background OD of blank well. The percent stimulation ratio (% SR) was calculated as under:

$$\%\text{SR}\;=\;\frac{\text{OD treated cells}\;-\;\text{OD untreated cells}}{\text{OD untreated cells}}\times 100$$

#### Nitrite assay

Basal as well as stimulated nitrite release was assayed in macrophage culture. Nitrite content was measured by the method of Ding et al. [48]. *In vitro* culture of macrophages and GS extract treatment were the same as described in subsection NBT reduction assay. Plate was incubated in CO_2_ atmosphere at 37°C for 24 h, centrifuged at 200xg, and supernatant was collected. Equal volume of supernatant and Griess reagent was mixed, and optical density (OD) was measured at 540 nm with the help of ELISA plate reader. OD was corrected by subtracting the background OD of blank well. Nitrite concentrations were extrapolated from calibration curve, linear regression [S1 Fig], constructed using sodium nitrite as a standard. The percent stimulation ratio (% SR) was calculated as under:

$$\%\text{SR}\;=\;\frac{\text{Nitrite content in treated cells}\;-\;\text{Nitrite content in untreated cells}}{\text{Nitrite content in untreated cells}}\times 100$$

#### Splenic lymphocyte proliferation assay

The lymphocyte proliferation was assessed colorimetrically based on tetrazolium salt (MTT) following the methods of Berridge et al. [49]. The colorimetric method, utilizing tetrazolium salts, has been an advantageous alternative method measuring lymphoproliferation [50]. Tetrazolium salt is reduced into a colored formazan product in the mitochondria of metabolically active cells. The quantity of formazan, as measured by the amount of absorbance at 570 nm, is directly proportional to the number of living cells in culture. Thus, quantifying the conversion of salt by mitochondrial dehydrogenases into colored formazan product provides a measure of cell number (not mitoses *per se*) during last hours of *in vitro* culture [51]. The accumulation of colored formazan is positively correlated with incorporation of ^3^H-thymidine into cellular DNA in the S-Phase of cell division during last hours of *in vitro* culture, which is a direct measure of blastogenesis under the conditions of mitogenic stimulation [52].

Splenic lymphocytes isolated from rat were assayed for untreated/ basal, mitogen- induced as well as GS extract stimulated lymphoproliferation. Splenic lymphocytes (10^6^cells per well) were seeded into well of flat- bottom 96 well culture plate. Mitogens were added into well; the final concentration was 5μg/ml for ConA and 10 μg/ml for PHA and LPS. GS extract (Final concentrations: 1, 5, 10, 20, 50, 100 and 200 μg/ml) was added into well with or without mitogen, and final volume was made 200μl/well with culture medium. Lymphocyte suspension with mitogen-free culture medium in well represented the untreated control proliferation. Lymphocyte cultures with different mitogens represented positive controls. Additional well containing 200 μl of culture medium served as blank. All assays were made in triplicate. Following incubation in humidified CO_2_ atmosphere at 37°C for 48 h, MTT (5 mg/ml) was added into each incubation well, and plate was again incubated for 4 h. After incubation, plate was centrifuged at 400xg for 10 min, the supernatant was aspirated, and formazan was dissolved in 100 μl of DMSO. Optical density (OD) was measured at 570 nm in ELISA plate reader. OD was corrected by subtracting the background OD of blank well. The percent stimulation ratio (% SR) was calculated as under:

$$\%\text{SR}\;=\frac{\text{OD treated cells}\;-\;\text{OD untreated cells}}{\text{OD untreated cells}}\times 100$$

### Statistical analysis

Data are presented as Mean ± SEM of triplicate determinations from three independent experiments. Data were analyzed by ANOVA followed by Duncan’s Multiple-Range test. Regression analysis was also performed to establish concentration-response relation, and EC_50_, effective concentration required for 50% stimulation, was extrapolated from linear regression equation [S2 Fig]. All statistical analyses were performed using Microsoft Excel and SPSS for Windows. All null hypotheses were tested at p<0.05.

## Results

### NBT reduction

**Fig 1:**
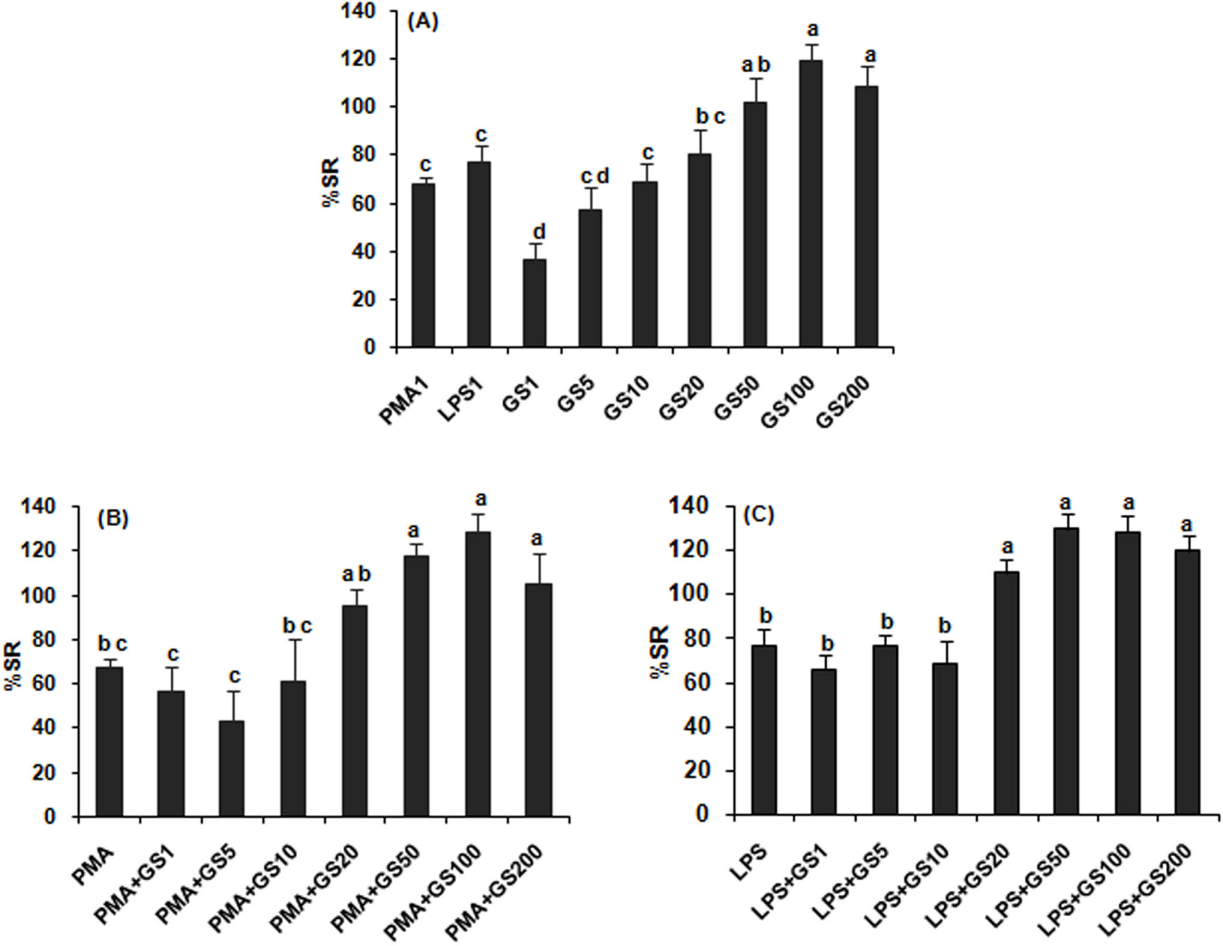
NBT reduction in rat peritoneal macrophages *in vitro* treated with *Gymnema sylvestre* leaf extract alone (A) and in combination with PMA 1μg/ml (B) and LPS 1μg/ml (C). GS1 –GS200 represent concentration of extract, 1–200μg/ml, respectively. Bars bearing same alphabet do not differ significantly. [Treatment effect ANOVA: F = 10.69 (8,18); p<0.05, (B) F = 7.57 (7,16); p<0.05, (C) F = 14.27 (7,16); p<0.05].

GS extract stimulated NBT reduction in a concentration dependent manner up to 50 μg/ml (R^2^ = 0.85, y = 0.74x–33.90; p<0.05), being maximal (120%) at 100 μg//ml, above which no further increased stimulation was observed (Fig 1A). The EC_50_ value was 3.10μg/ml. The stimulation of NBT reduction was significantly (p<0.05) higher at 50, 100, 200μg/ml extract concentration than that caused by standard immunoelicitor, PMA or LPS; while 5, 10 and 20 μg/ml, showed stimulatory effect similar to that of LPS or PMA; and 1 μg/ml extract, less than that. When macrophages incubated with extract in presence of PMA or LPS, a higher level NBT dye reduction, though insignificant, occurred at each concentration. Maximal NBT reduction was observed at 50, 100 and 200 μg/ml extract concentration in presence of PMA or LPS; while 1, 5, 10 and 20μg/ml showed as much reduction as shown by PMA (Fig 1B) or LPS alone (Fig 1C).

### Nitrite release

**Fig 2:**
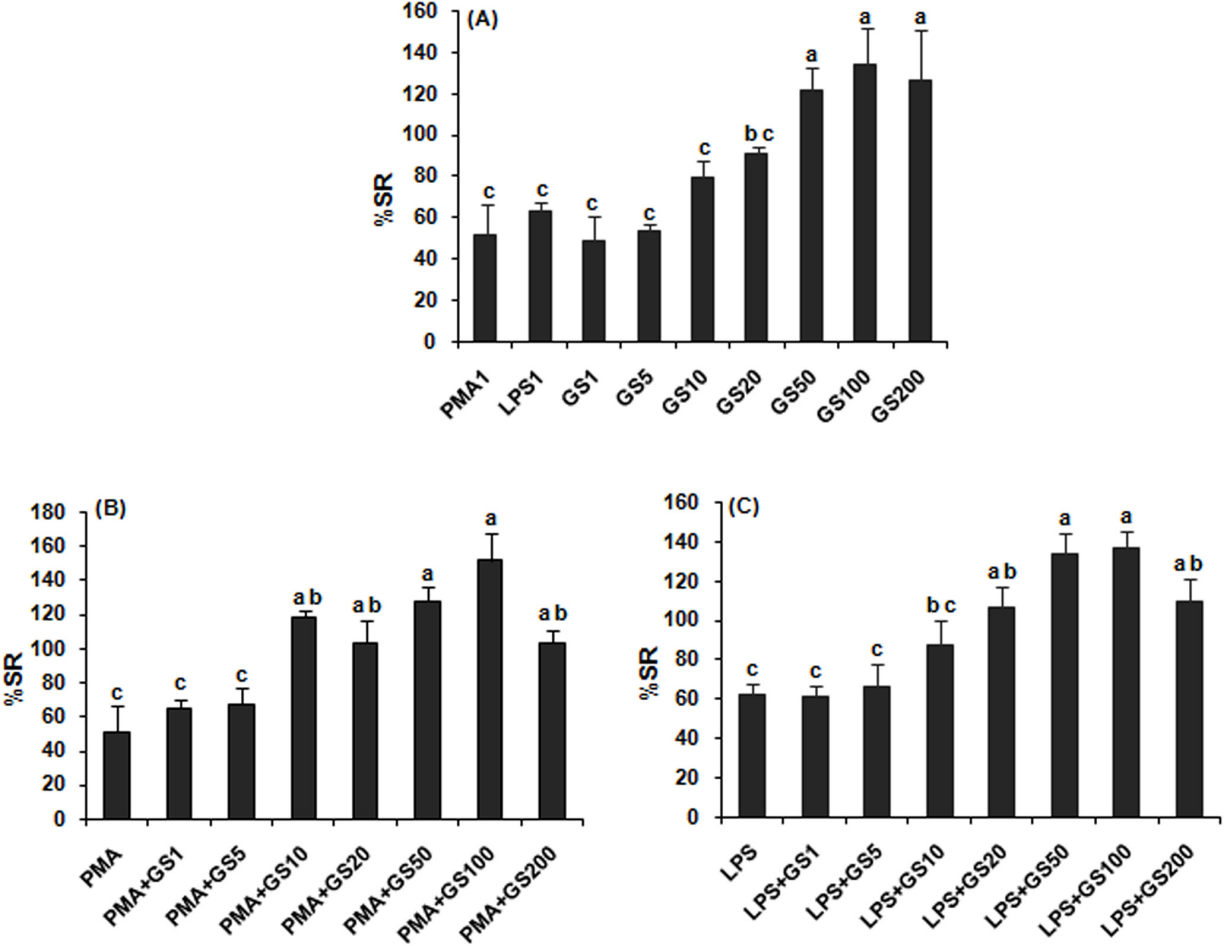
Nitrite release in rat peritoneal macrophages *in vitro* treated with *Gymnema sylvestre* leaf extract alone (A) and in combination with PMA 1μg/ml (B) and LPS 1μg/ml (C). GS1 –GS200 represent concentration of extract, 1–200μg/ml, respectively. Bars bearing same alphabet do not differ significantly. [Treatment effect ANOVA: (A) F = 7.10 (8, 18); p<0.05, (B) F = 5.05 (7, 16); p<0.05, (C) F = 10.29 (7, 16); p<0.05].

Nitrite release in macrophage culture was stimulated by 49, 54, 79, 91, 122, 134, and 126%, when cultured with 1, 5, 10, 20, 50, 100 and 200 μg/ml GS extract, respectively, as compared to LPS (62%) or PMA (51%). The stimulation in nitrite release was concentration dependent up to 50 μg/ml (R^2^ = 0.91; y = 0.64x–28.25; p<0.01: and EC_50_ value extrapolated from the linear equation was 3.75μg/ml), no further significant stimulation was recorded at 100 or 200 μg/ml (Fig 2A). The stimulatory effect of GS extract significantly (p<0.05) exceeded at 20, 50, 100 and 200 μg/ml concentrations than that of PMA or LPS; while lower concentrations 1, 5 and 10μg/ml, had effect identical to that of PMA or LPS. When macrophage cultures treated with GS extract in presence of PMA or LPS, 10 to100μg/ml concentrations showed higher level of stimulation, though non- significant, than GS extract alone, but significant than PMA (Fig 2B) or LPS (Fig 2C).

### Splenic lymphocyte proliferation

**Fig 3:**
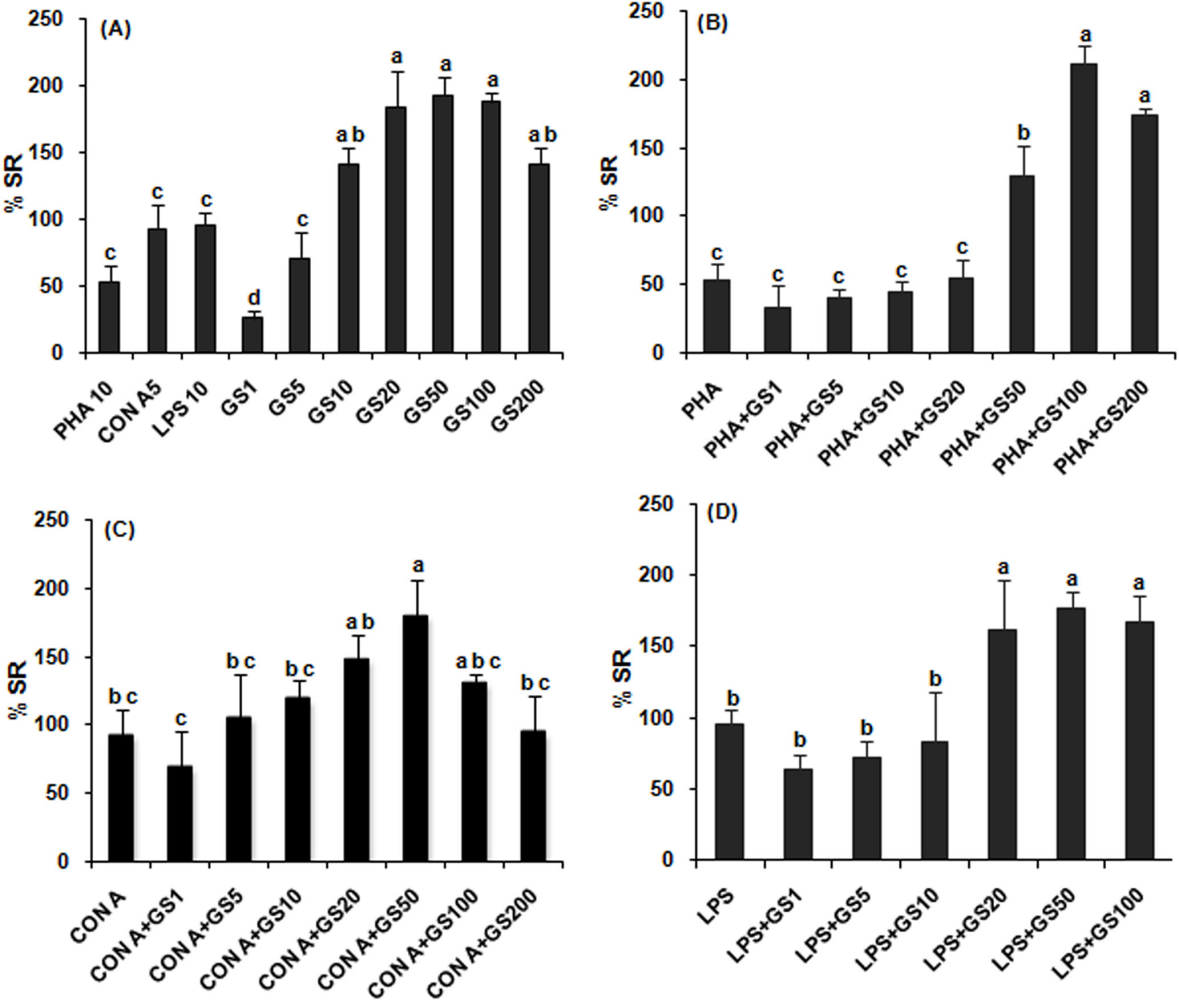
Proliferation of splenic lymphocytes *in vitro* treated with *Gymnema sylvestre* leaf extract alone (A) and in combination with PHA 10 μg/ ml (B), ConA 5 μg/ ml (C) and LPS 10 μg/ml (D). GS1 –GS200 represent concentration of extract, 1–200μg/ml, respectively. Bars bearing same alphabet do not differ significantly. [Treatment effect ANOVA: (A) F = 10.58 (9, 20); p<0.05, (B) F = 27.28(7, 16); p<0.05, (C) F = 2.66 (7, 16); p<0.05; (D) F = 5.26 (6, 14); p<0.05].

Effect of GS extract on splenic lymphocyte proliferation is presented in Fig 3. The proliferative response was stimulated by 26, 71, 141, 184, 193, 187 and 141% in cultures treated with 1, 5, 10, 20, 50, 100 and 200 μg/ml GS extract, respectively, as compared to mitogen-stimulated proliferative responses (PHA, 53%; ConA, 93% and LPS, 96%).The stimulatory effect was concentration dependent up to 20 μg/ml (R^2^ = 0.93; y = 0.11x–2.82; p<0.05: and EC_50_ value extrapolated from the linear equation was 2.68μg/ml); no further enhanced stimulation was observed at 50 and 100 μg/ml concentrations, rather decreased at 200 μg/ml (Fig 3A). When GS extract was combined with PHA in cultures, 50 to 200 μg/ml concentrations showed higher level response than PHA alone, being maximal at 100μg/ml; while the lower concentrations, similar level response (Fig 3B). Proliferative response was significant (p<0.05) and maximal in lymphocyte culture treated with 50 μg/ml GS extract in presence of another T-cell mitogens, ConA, but decreased when treated with higher concentrations (Fig 3C). When the splenic lymphocyte cultures treated with GS extract along with B-cell mitogens (LPS), 20–100 μg/ml concentrations enhanced the proliferative response significantly (p<0.05) and maximally of identical level, as compared to LPS-stimulated one (Fig 3D).

### Analysis of extract

**Fig 4:**
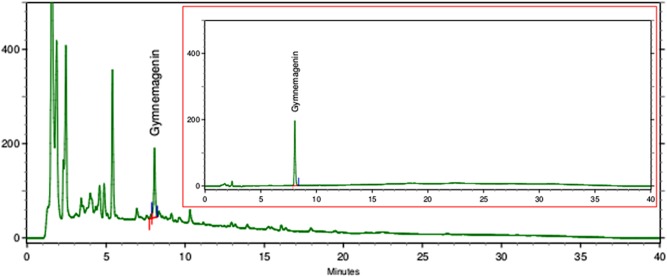
HPLC chromatograms of *Gymnema sylvestre* leaf extract and reference standard, gymnemagenin, as insert within image.

The identification and quantification of gymnemic acid in leaf extract was done by comparing the retention time and peak area of sample extract with that of the reference standard. The results emanated from HPLC analysis of leaf extract are summarized in Table 1. Fig 4 shows chromatograms of reference standard gymnemagenin, with a unique λ_max_ at 205 nm, and of *G*. *sylvestre* leaf extract, hydrolysed according to the method described. Chromatogram of leaf extract showed a sharp and symmetrical peak that was identical to that of standard gymnemagenin. Gymnemic acid content in leaf extract was found to be 2.40% dry weight.

**Table 1:** HPLC parameters of reference standard and leaf extract.

	Retention time (min)	Peak Area	Gymnemic acid (%w/w)
Gymnemagenin	8.056	21780487	-
Leaf extract	8.061	16733596	2.40

## Discussion

*G*. *sylvestre* has been emerged as a potential panacea for the management of diabetes. Many studies on *G*. *sylvestre* have demonstrated that it may exert its anti-diabetic effect via a number of pathways [34]. In a study by Shanmugasundaram et al [36], oral treatment of streptozotocin-induced diabetic rats with *G*. *sylvestre* extracts (20 mg/day/rat) normalized the fasting glucose level and rise in insulin level by repair/regeneration of endocrine pancreas. Support for the efficacy of *G*. *sylvestre* in management of both type 1 and type 2 diabetes comes from two earlier controlled, open-label clinical trials, where *G*. *sylvestre* extract (400 mg /day) has been given orally to 27 patients with type 1 diabetes as supplement to insulin therapy for 16–18 months [53] and to 22 patients with type 2 diabetes as supplement to conventional drug therapy [54]. During *G*. *sylvestre* extract supplementation, patients showed significant reduction in blood glucose, glycosylated haemoglobin and glycosylated plasma proteins, and conventional drug dosage could be reduced. In another study, oral administration of a novel extract, OSA^®^, (1mg/day) for 60 days is reported insulinotropic and anti-hyperglycaemic in a small cohort of type 2 diabetics [40]. The same novel extract improves glucose tolerance *in vivo* in mice and stimulates insulin secretion from isolated mouse islets *in vitro* [41] and from MIN6 β-cell line and isolated human islets of Langerhans *in vitro* [39].

This study for the first time evaluated the effect of GS extract on immune activities of rat macrophages and splenic lymphocytes. Macrophages, comprising the major cellular components of the innate immune system, perform phagocytosis of pathogens and *de novo* production of a range of ephemeral, reactive oxygen species (ROS), such as super-oxide anion (O_2_^–^) with powerful microbicidal (cytotoxic) activity during the respiratory burst [55]. Respiratory burst function resulting in the release of ROS is one of the key mechanisms–the oxygen-dependent defense mechanism–of the innate immune system. The respiratory burst can be elicited, upon suitable stimulation by soluble components, namely PMA, lectins, LPS or by particulate phagocytic stimulus, such as zymosan. To this end, the production of ROS by peritoneal macrophages was investigated using a quantitative microplate assay of the reduction of NBT. The NBT test involves assessment of the ability of macrophages to reduce the yellow soluble redox dye NBT to form blue formazan, a water insoluble material that precipitates intracellularly. The degree of production of formazan provides a quantitative measurement of superoxide anion (O₂^-^).

Nitric oxide (NO) is another effecter molecule of macrophage cytotoxicity. It is produced from L-arginine by the enzyme NOS. Soon after production, NO decomposes to other nitrogen oxides, popularly known as RNI. So, nitrite was also assayed as a marker of macrophage cytotoxicity in this study. In laboratory, the quantification of nitrite release and NBT reduction in macrophage and of mitogen-induced lymphoproliferative response are the most widely used *in vitro* evaluation of functional assessment of cellular arm of immune system: the greater the *in vitro* response, the more effective *in vivo* immune response is the implicit assumption in performance of these assays.

Following GS extractstimulation (1–10 μg/ml), macrophage reactivity, ROS and NO production was identical to PMA or LPS stimulation, but enhanced more than that of PMA or LPS at higher concentrations of extract (50–200 μg/ml). When GS extract combined with PMA or LPS in macrophage culture, a higher level of stimulation occurred, though insignificant, as compared to GS treatment alone. The results suggest that GS extract may be able to induce respiratory burst. ROS and NO production by macrophages following GS extract stimulation may provide various activities (antimicrobial, antitumor, antiviral, etc.) under specific conditions *in vivo*. Antitumor-cytotoxic activity of isolated saponin on HeLa cells [56] and water soluble polysaccharides fraction on AGS, SCG and U937 cells [57] from *G*. *sylvestre* has been reported elsewhere. The results of the present study are in conformity with the effects of other herbal plant extracts on ROS and NO production in macrophages [1, 5, 9–11, 14, 17,] and on splenic lymphocyte proliferation in rat/mouse [2, 6–8, 14]. Mitogens used in this study, Con A and PHA, are specifically mitogenic for T-cell; while LPS, for B-cell. We found that ConA- and LPS-induced proliferative responses were higher than PHA-induced one. Due to identical effects on splenic lymphoproliferation, as measured by MTT assay, with or without mitogens, GS extract active component, gymnemic acid, is involved equally in T- and B-cell proliferation stimulation.

The present study demonstrates that GS extract stimulates macrophage reactivity significantly, increasing the level of activity even higher but insignificantly when combined with PMA or LPS. Similarly, GS extract stimulates splenic lymphoproliferation. GS extract concentrations used in this study were not toxic to rat macrophages and lymphocytes. EC_50_ values as ascertained were 3.10, 3.75 and 2.68μg/ml for NBT reduction, nitrite release and lymphocyte proliferation, respectively, suggesting that a lower concentration of GS extract is effective to stimulate proliferation in lymphocytes, but a higher one is required to stimulate superoxide anion and NO production in macrophages.

The method of sample preparation is important and significant with respect to the analysis of herbal drugs. Also, quality control and standardization of herbal drug are important issues in herbal drug development. WHO regulatory guidelines [58] recommend use of a marker based as well as fingerprint approach for standardization of herbal drug. In this study, a marker based approach was used to standardize the traditionally important herbal medicine, G.sylvestre. Amongst the complex mixture of biologically active compounds, gymnemagenin can be used as an analytical marker in order to assess the quality of samples of *G*.*sylvestre* obtained from different sources. HPLC characterization of leaf extract in the present study allowed the reliable quantification of gymnemic acid with good resolution from other constituents of G. sylvestre.

It seems that GS extract, prepared in this study, contains active components, gymnemic acids that not only stimulate superoxide and NO production by macrophages and lymphocyte proliferation, but also act synergistically with elicitors, PMA or LPS, and the mitogens (ConA, PHA, and LPS). The GS extract may provide a second signal for synergistic induction of superoxide anion and NO production in macrophages and the proliferation of lymphocytes. Thus, the present study demonstrates that GS extract not only improves the metabolic and endocrine disorders in diabetes, as discussed above, but also can ameliorate the depressed immunity in diabetes, that has been reported in earlier studies [31–33].Molecular approaches to define mechanistic basis of immunomodulation by GS extract and its*in vivo* immunomodulatory activity in normal and diabetic rat model would merit further attention.

## Conclusions

Present study suggests that the extract of *G*. *sylvestre* leaf contains the active compounds, Gymnemic acid, which has modulatory effects on rat immune system. The traditional knowledge on the use of *G*. *sylvestre* in treating diabetes was scientifically supplemented for its immunomodulatory properties. We believe that *G*. *sylvestre* leaf extract could re-establish the macrophage reactivity and the lymphocytes proliferation capacity, and thereby, can restore the innate immune functions.

## Supporting Information

S1 ARRIVE Checklist NC3Rs ARRIVE Guidelines checklist. (PDF)

S1 Fig Nitrite standard calibration curve constructed following linear regression analysis. (TIF)

S2 Fig *Gymnema sylvestre* leaf extract (GS) concentration–response (% SR) curve constructed following linear regression analysis. A- NBT reduction; B- Nitrite release; C- Splenic lymphoproliferation
(TIF)
