# Supplementary figures and images for: Immunomodulatory Effect of Gymnema sylvestre (R.Br.) Leaf Extract: An In Vitro Study in Rat Model
Source: PLoS One. 2015 Oct 16;10(10):e0139631. doi: 10.1371/journal.pone.0139631 (PMC4608767; doi:10.1371/journal.pone.0139631)

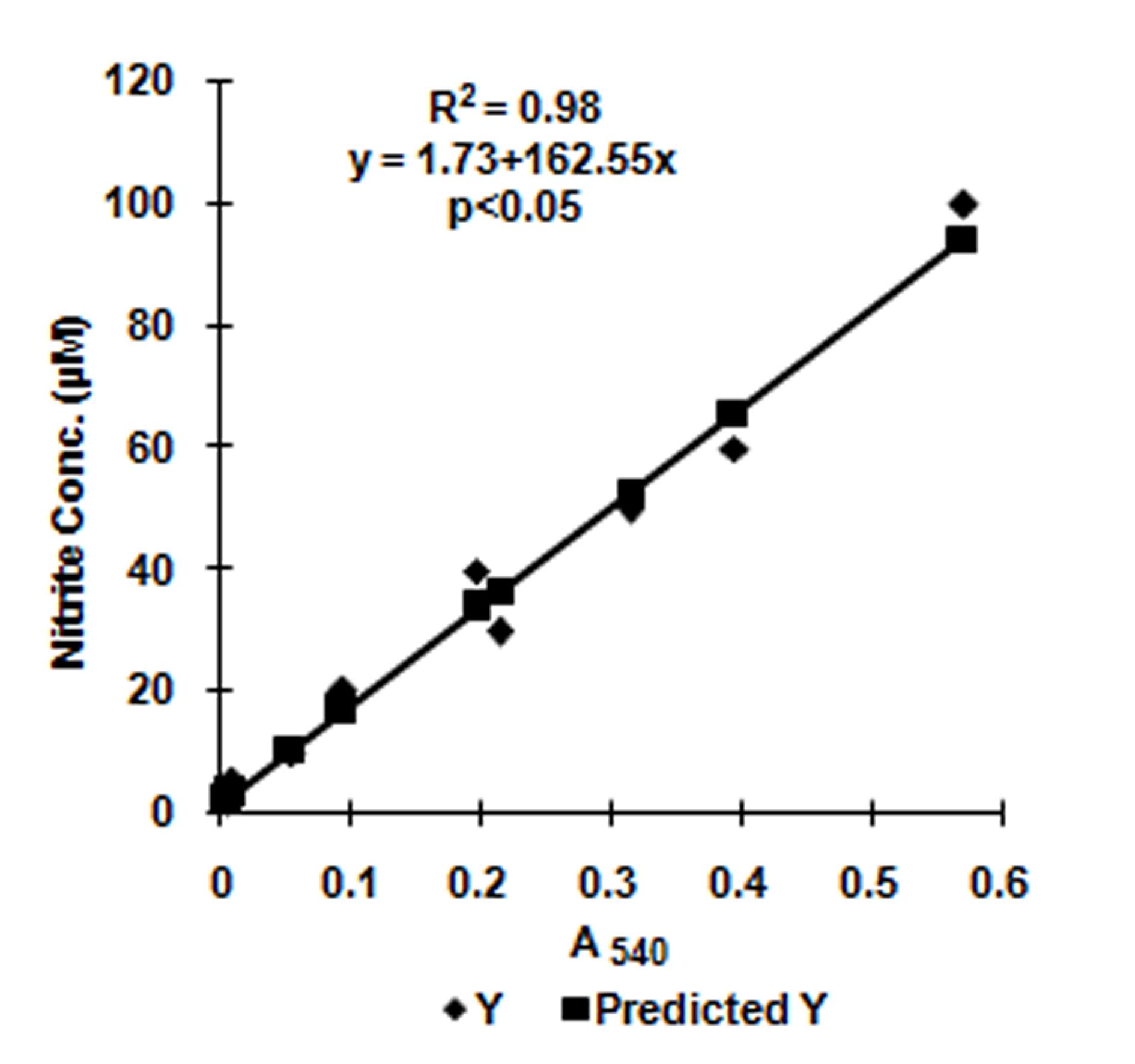

Supplement: S1 Fig — (TIF) [file pone.0139631.s002.tif]

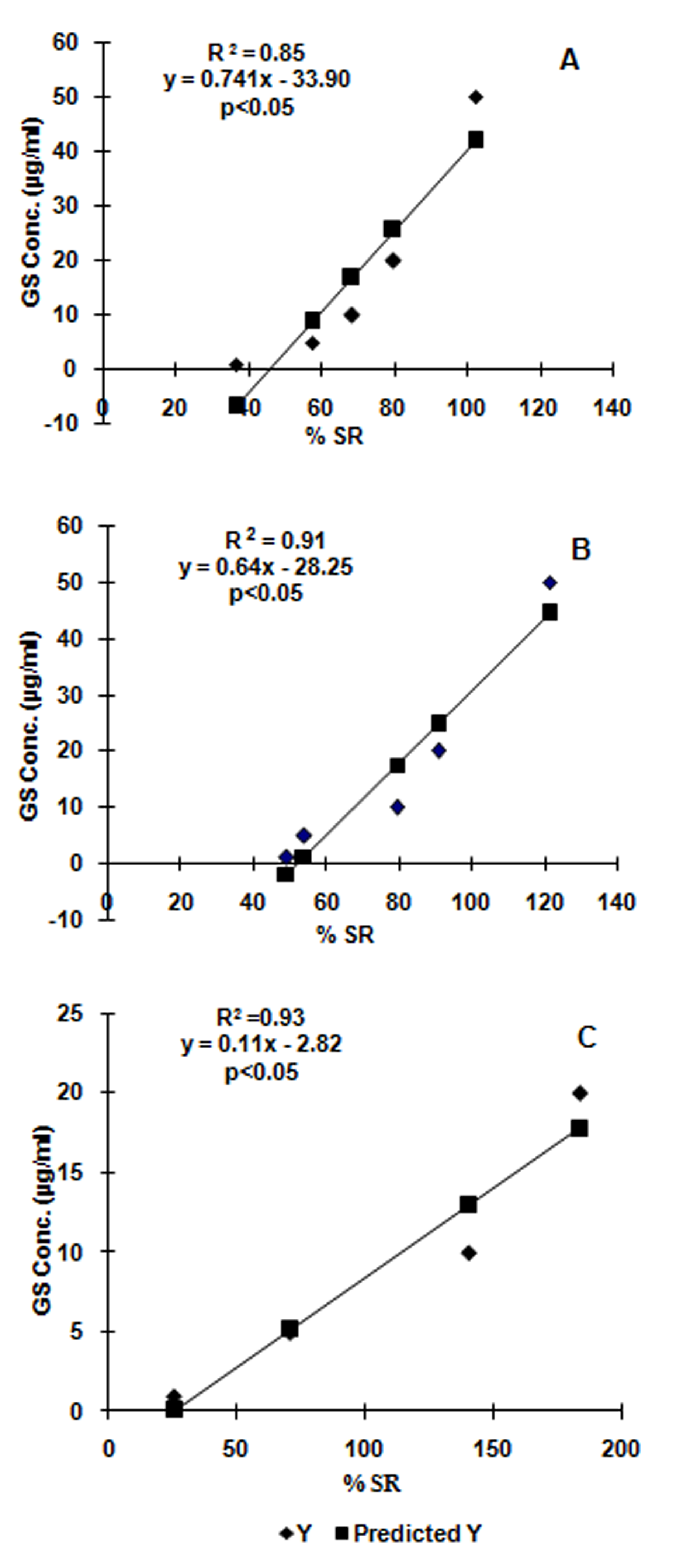

Supplement: S2 Fig — A- NBT reduction; B- Nitrite release; C- Splenic lymphoproliferation (TIF) [file pone.0139631.s003.tif]
